# Evaluation of a Novel Enteral Phosphorus Therapy with Enteral Nutrition during a National Intravenous Sodium Phosphate Shortage

**DOI:** 10.3390/nu16091394

**Published:** 2024-05-06

**Authors:** Tinia D. Harris, Julie E. Farrar, Saskya Byerly, Dina M. Filiberto, Roland N. Dickerson

**Affiliations:** 1 Department of Pharmacy, Regional One Health, Memphis, TN 38103, USA; 2 Department of Clinical Pharmacy and Translational Science, University of Tennessee Health Science Center, Memphis, TN 38163, USA; 3 Department of Surgery, University of Tennessee Health Science Center, Memphis, TN 38163, USA

**Keywords:** phosphorus, calcium, refeeding syndrome, critical illness, trauma, drug shortages, enteral nutrition

## Abstract

The purpose of this study was to evaluate the efficacy and safety of intragastric administration of small volumes of sodium enema solution containing phosphorus as phosphorus replacement therapy in critically ill patients with traumatic injuries who required continuous enteral nutrition. Adult patients (>17 years of age) who had a serum phosphorus concentration <3 mg/dL (0.97 mmol/L) were evaluated. Patients with a serum creatinine concentration >1.4 mg/dL (124 µmol/L) were excluded. Patients were given 20 mL of saline enema solution intragastrically, containing 34 mmol of phosphorus and mixed in 240 mL water. A total of 55% and 73% of patients who received one (*n* = 22) or two doses (*n* = 11) had an improvement in the serum phosphorus concentration, respectively. The serum phosphorus concentration increased from 2.5 [2.1, 2.8] mg/dL (0.81 [0.69, 0.90] mmol/L) to 2.9 [2.2, 3.0] mg/dL (0.94 [0.71, 0.97 mmol/L) for those who received two doses (*p* = 0.222). Excluding two patients with a marked decline in serum phosphorus by 1.3 mg/dL (0.32 mmol/L) resulted in an increase in the serum phosphorus concentration from 2.3 [2.0, 2.8] mg/dL (0.74 [0.65, 0.90] mmol/L) to 2.9 [2.5, 3.2] mg/dL (0.94 [0.81, 1.03] mmol/L; *n* = 9; *p* = 0.012). No significant adverse effects were noted. Our data indicated that intragastric phosphate administration using a small volume of saline enema solution improved the serum phosphorus concentrations in most patients.

## 1. Introduction

During a recent national intravenous sodium phosphate shortage, intravenous potassium phosphate was used whenever possible in lieu of sodium phosphate. However, potassium phosphate may not be a preferred salt form for phosphorus repletion therapy when the patient is normokalemic or hyperkalemic. Critically ill patients with traumatic injuries have high phosphorus demands, particularly those receiving nutrition therapy [1]. Repletion of hypophosphatemia is challenging, especially when performed by the enteral route, as only 40% to 70% of enteral phosphorus is absorbed [2,3,4] and high enteral phosphorus doses are associated with diarrhea. As a result, intravenous phosphate preparations are often preferred for critically ill patients with multiple traumatic injuries who are receiving nutrition therapy [1].

Given the potential for worsened clinical outcomes during drug shortages, it is imperative to find safe and efficacious alternative solutions [5]. The intent of this study was to evaluate the safety and efficacy of temporary enteral administration of a small amount of diluted saline enema solution containing phosphorus for the treatment of hypophosphatemia in enterally fed patients with severe traumatic injuries during a national intravenous sodium phosphate shortage.

## 2. Materials and Methods

Adult patients (>17 years of age) admitted to the trauma intensive care unit (TICU) who required continuous intragastric enteral nutrition (EN) and had a serum phosphorus concentration <3 mg/dL (0.97 mmol/L) during a national intravenous sodium phosphorus shortage were evaluated. Patients who had a serum potassium concentration >3.9 mEq/L (3.9 mmol/L) or were currently being given potassium chloride for hypokalemia were excluded. The other exclusion criteria were patients with a serum creatinine concentration >1.4 mg/dL (124 µmol/L) or those who required hemodialysis or renal replacement therapy.

Patients were given short-term administration of a fixed, weight-independent dose of 20 mL of saline enema solution (C.B. Fleet Company, Inc., Lynchburg, VA, USA). A second dose could be given 4 to 6 h later as per the discretion of the Nutrition Support Service (NSS). Saline enema solution contains 19 g of monobasic sodium phosphate (NaH_2_PO_4_) and 7 g of dibasic sodium phosphate (Na_2_H_3_PO_5_) per 118 mL. A total of 20 mL was extracted by pharmacy personnel and delivered to the patient care unit in a small plastic container. This 20 mL of the enema solution will provide about 34 mmol of elemental phosphorus and 41 mEq (41 mmol) of sodium, which is similar to the electrolyte content of intravenous sodium phosphate. The instructions for use provided to nursing personnel were to dilute the 20 mL of saline enema solution with 240 mL of water prior to intragastric administration. Its osmolarity is about 1750 mOsm/L when undiluted, which is reduced to about 135 mOsm/L when diluted with water.

Although it is common practice to determine the appropriate intravenous phosphorus dose based on the measured body weight or adjusted weight and serum phosphorus concentration [1,3], one or two fixed enteral doses of phosphorus (34 mmol or 68 mmol, respectively) were given for ease of standardization and to reduce errors in its preparation. The dosing weight was expressed based on the actual body weight unless the patient was obese (BMI > 29.9 kg/m^2^); then, an adjusted weight was determined for drug and electrolyte dosing. The adjusted dosing weight for patients with obesity was calculated by ideal body weight (IBW, kg) + [(current body weight(kg) − IBW (kg)) × 0.4] [6].

Patients were retrospectively identified from the electronic medical health record and the NSS monitoring records. The hospital’s electronic medical records were accessed to obtain pertinent laboratory, nutritional, and demographic variables. Details were recorded for the day of the enteral phosphorus therapy and the day thereafter. Serum laboratory tests were ordered either by the primary trauma service or the NSS and performed by the hospital laboratory as part of the patients’ routine clinical care. Daily serum laboratory determinations were drawn between 0000 and 0100 h. The selection of the enteral feeding formulation, assignment of infusion rates, and prescribed electrolyte supplementation was conducted by members of the NSS in conjunction with the primary trauma service. EN was initiated at 20 to 40 mL/h and advanced by 20 to 40 mL/h daily as tolerated, based on the gastric feeding tolerance, extent of hyperglycemia, and electrolyte perturbations, until the desired goal rate was achieved, as previously described [7]. Boluses of modular liquid protein supplement were given via the intragastric tube when necessary to meet protein goals.

The efficacy of the enteral phosphorus therapy was evaluated by the difference in serum phosphorus concentration prior to and after phosphorus therapy. The safety was evaluated by the presence of emesis, diarrhea, or a significant fall in the serum ionized calcium concentration (iCa) greater than 0.1 mmol/L or the presence of moderate or severe hypocalcemia (iCa < 1 mmol/L) within 24 h after the phosphorus therapy was given. Diarrhea was defined as >2 liquid stools/day or >300 mL/day, or by the placement of a fecal management system by nursing personnel [8].

Continuous data were given as the median [25th, 75th percentile]. Chi square analysis or Fisher’s exact test was used to compare nominal data. Continuous data were analyzed using the Wilcoxon rank sum test for independent variables and the Wilcoxon signed rank test for paired variables. Statistical significance was defined as a *p* value < 0.05. Trending increases in the serum phosphorus concentration were assessed for a potential type II error via power analysis using a power (ß) of 0.8 and *p* < 0.05. The study was approved by the University Investigational Review Board and the hospital’s Office for Medical Research. The requirement for informed consent was waived.

## 3. Results

Thirty-three patients who received a dose of saline enema solution during continuous EN were evaluated. Most patients were male (70%), Black (61%), ventilator-dependent (97%), admitted due to a motor vehicle collision (70%), and survived (82%). The patients were generally younger (46 [29, 62] years of age), and they were early in the course of their EN therapy (3 [2, 7] days) and TICU duration of stay (5 [4, 10] days). All the patients had good kidney function, as evidenced by a serum creatinine concentration of 0.8 [0.7, 0.9] mg/dL (71 [62, 80] µmol/L). The patients demonstrated a significant systemic inflammatory response with a markedly elevated serum C-reactive protein concentration 23 [15.6, 33.2] mg/dL (230 [156, 332] mg/L) and decreased serum prealbumin concentration 9.0 [6.0, 13.0] mg/dL (90 [60, 130] mg/L). The TICU and hospital length of stays were 14 [11, 18] days and 25 [19, 41] days, respectively. Other demographic data can be found in Table 1.

**Figure 1:**
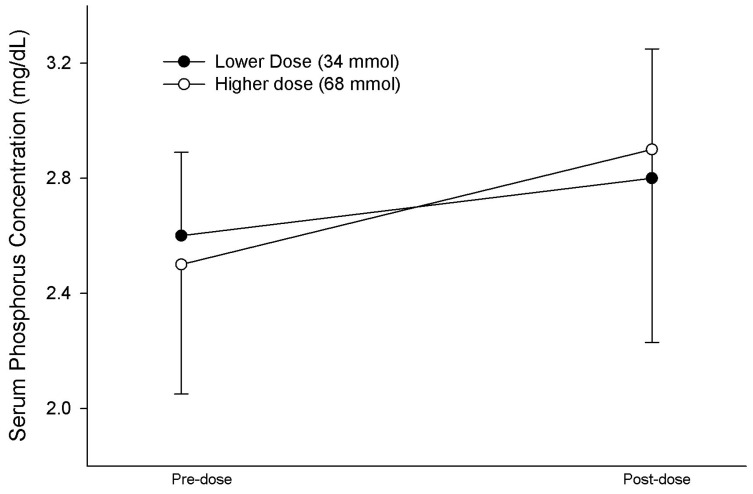
Pre-dose and post-dose serum phosphorus concentration response to 34 mmol and 68 mmol of phosphorus administered intragastrically. No statistically or clinically relevant differences in the serum phosphorus concentration were noted between dosage groups or pre- vs. post-dose for each group. The serum phosphorus concentration in mmol/L can be calculated by 0.323 X serum phosphorus concentration (mg/dL). Data are given as the mean ± S.D.

A total of 22 patients received a single 34 mmol dose. The serum phosphorus concentration tended to increase from 2.6 [2.4, 2.8] mg/dL (0.84 [0.78, 0.90] mmol/L) to 2.8 [1.9, 3.3] mg/dL (0.90 [0.61, 1.07] mmol/L), but the difference was statistically insignificant (*p* = 0.430). A total of 12 patients (55% of the population) had an increase in the serum phosphorus concentration, but 5 patients had a significant decline in the serum phosphorus concentration to <2 mg/dL (0.65 mmol/L), ranging from 1.6 to 1.9 mg/dL (0.52 to 0.61 mmol/L). Moreover, 11 patients received 2 doses for a total of 68 mmol. Eight patients (73% of the population) had an improvement in the serum phosphorus concentration. The serum phosphorus concentration increased from 2.5 [2.1, 2.8] mg/dL (0.81 [0.68, 0.90] mmol/L) to 2.9 [2.2, 3.0] mg/dL (0.94 [0.71, 0.97] mmol/L; *p* = 0.222). Power analysis of the data indicated that 60 patients were necessary to achieve statistical significance. A total of 2 patients (18% of the population) in the higher dosage group had a significant decline in serum phosphorus by 1.3 mg/dL (0.42 mmol/L) from the baseline concentration. The omission of these two patients, whose serum phosphorus declined from 2.6 (0.84 mmol/L) to 1.3 mg/dL (0.42 mmol/L) and from 2.9 (0.94 mmol/L) to 1.6 mg/dL (0.52 mmol/L), resulted in an increase in the serum phosphorus concentration from 2.3 [2.0, 2.8] mg/dL (0.74 [0.65, 0.90 mmol/L) to 2.9 [2.5, 3.2] mg/dL (0.94 [0.81, 1.03 mmol/L) in the remaining 9 patients (*p* = 0.012). The responses and the factors that may influence the phosphorus response for each dosage group are summarized in Table 2. The changes in the serum phosphorus concentration for each dosage group are shown in Figure 1.

**Figure 2:**
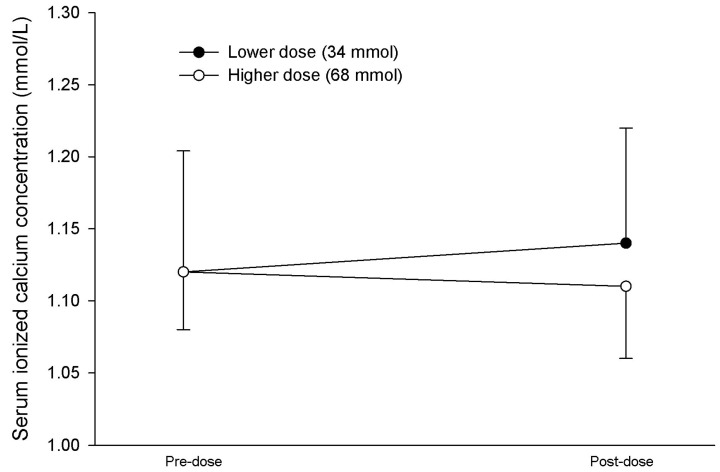
Pre-dose and post-dose serum ionized calcium concentration response to 34 mmol and 68 mmol of phosphorus administered intragastrically. No statistically or clinically relevant differences in the serum ionized calcium concentration were noted between dosage groups or pre- vs. post-dose for each group. Data are given as the mean ± S.D.

No significant differences were noted in iCa at either dose (Table 2). However, one patient had a significant fall in the serum iCa from 1.17 to 1.05 mmol/L and another experienced a fall in the serum iCa from 1.06 to 0.99 mmol/L in the higher dosage group. No adverse effects of hypocalcemia were noted in the patients’ medical records. Data regarding the changes in the serum iCa concentration for each dosage group are provided in Figure 2.

The differences in those who responded to enteral phosphorus therapy versus those who worsened could not be explained by the differences in carbohydrate, caloric, or insulin intakes, arterial pH, presence of traumatic brain injury or other clinical or laboratory parameters (Table 3).

Three patients, all from the lower dosage group, experienced diarrhea. One of these patients received multiple doses of sorbitol-containing acetaminophen liquid, while the other two patients were receiving concurrent laxatives for prophylaxis of opioid-induced constipation. Two patients among these patients were also receiving intravenous broad spectrum antibiotic therapy (cefepime). A fecal management system was already in place prior to the enteral phosphorus administration in one patient. None of the patients experienced emesis and the highest gastric residual volume recorded for those with potential intolerance due to diarrhea was 180 mL on the day the saline enema solution was given.

## 4. Discussion

Our data indicated that intragastric phosphate administration using saline enema solution containing phosphorus improved the serum phosphorus concentration in most patients who required continuous enteral nutrition. Those who received two doses of phosphate responded better than those who received one dose, but these data were not statistically significant. Adverse effects, particularly diarrhea, were minimal and complicated by the coadministration of sorbitol-containing liquid medications, laxatives, and broad-spectrum antibiotic therapy. However, the serum phosphate response to intragastric phosphate administration was variable, with about half of the patients receiving the single dose and three-fourths of the higher dose group demonstrating an improvement in the serum phosphorus concentration. Critically ill patients with traumatic injuries who receive this therapy will require vigilant monitoring and reassessment.

Untreated severe hypophosphatemia can lead to the impaired muscle contractility of the diaphragm [9] and heart [10], a shift to the left in the oxyhemoglobin dissociation curve [11], seizures [12], and even death [13]. Patients were evaluated after a median of 2 days of receiving EN. Decreases in the serum phosphorus concentration are common in critically ill trauma patients who receive nutrition, particularly in the early stages of nutrition therapy [3]. Hypophosphatemia in this population may be the result of multiple factors, including refeeding syndrome [3], ß adrenergic catecholamine stimulation [14], infection [15], hyperventilation [16], carbohydrate intake and insulin [16], traumatic brain injury [17], and increased energy expenditure [18]. As a result, clinicians are often very proactive with aggressive phosphorus therapy in anticipation of a significant fall in the serum phosphorus concentration during the early stages of EN for critically ill trauma patients [1,17].

In late 2021 through early 2022, a national shortage of intravenous sodium phosphate occurred in the United States. Because critically ill patients with traumatic injuries require high repletion doses of phosphorus [1,17], we have preferred intravenous phosphorus administration as it is 100% bioavailable as opposed to 40% to 70% bioavailable with enteral administration [2,3,4]. In addition, intravenous phosphorus administration avoids the potential adverse effect of large doses of enteral phosphorus, which can cause diarrhea. Unfortunately, unlike a previous national shortage, intravenous sodium glycerophosphate was no longer available as it was only approved in the short term by the U.S. Food and Drug Administration as an alternative source of intravenous sodium phosphate. However, sodium glycerophosphate is still available in other countries outside of the United States and can be used as an alternative source of intravenous potassium-free phosphorus therapy. It is available in single 20 mL dose vials containing 20 mmol of organic phosphorus and 40 mEq (40 mmol) of sodium. As a result, potassium phosphate was used whenever possible; however, this salt form was not always preferable in certain conditions, such as hyperkalemia, or for patients previously given aggressive potassium chloride therapy. The lack of an intravenous form of sodium phosphate necessitated a trial of enteral phosphate. At our institution, only sodium phosphate tablets containing 250 mg or 8 mmol of phosphate were available at that time. The crushing of several tablets per administration once or twice daily per small-bore feeding tube was not considered a preferred option due to the high potential for tube clogging and significant nursing drug preparation and administration time. Other oral phosphate options, such as sodium phosphate capsules (250 mg or 8 mmol to be opened and the contents mixed with water), were also not available at our institution at that time. Sodium phosphate capsules are also limited in that the manufacturer recommends that the content of one sodium phosphate capsule be mixed in at least 120 mL of water. Thus, the final volume (e.g., 480 mL) may not be well tolerated in addition to enteral delivery of other medications, modular protein boluses, and continuous EN.

As a short-term temporary solution, we opted to use a small volume of diluted commercially available saline enema solution to be given concurrently with the continuous EN. A total of 20 mL of the enema solution provides about 34 mmol of elemental phosphorus. Since its osmolarity is approximately 1750 mOsm/L when undiluted and administration of hypertonic medications is known to cause adverse gastrointestinal effects [19] such as dumping syndrome, we diluted 20 mL of saline enema solution with 240 mL of water to reduce its osmolarity to about 135 mOsm/L. In addition, only intragastric administration of this preparation was allowed and post-pyloric administration was avoided.

Patients were empirically given one or two doses at the discretion of the NSS. Only about half of the patients given a single dose had an improvement in the serum phosphorus concentration; however, about three-fourths of those given two doses had an improvement in the serum phosphorus concentration. Unfortunately, the clinical features of a lack of responsiveness could not be ascertained (Table 3). This tempered therapeutic response can potentially be explained by multiple factors. Variability in bioavailability has been documented with oral phosphate absorption [2,3,4]. How much of the elemental phosphorus from the enema solution that is bound to the EN is unknown. Over 50% of critically ill patients with traumatic injuries have augmented renal clearance, as defined by a creatinine clearance greater than 150 mL/h [20]. This high incidence of augmented renal clearance may also partially explain why critically ill trauma patients require high doses of phosphorus as well as other intracellular electrolytes during nutrition therapy [1,17]. Another potential explanation is that critically ill trauma patients have a variable renal phosphate threshold concentration (TmP/GFR) of about 3.1 to 3.3 mg/dL (1.00 to 1.07 mmol/L), whereby higher doses may result in temporarily greater serum phosphorus concentrations but ultimately lead to increased fractional excretion of phosphorus for some patients [21]. It is unknown whether higher enteral doses will be more effective in improving the serum phosphorus concentrations without causing adverse effects (e.g., diarrhea, hypocalcemia) in this population.

Acute phosphate nephropathy, resulting in acute kidney injury, has been reported in association with ingestion of oral phospho-soda solution containing about 180 mmol of phosphorus [22]. Thus, care must be taken to avoid excessive doses, particularly in older patients greater than 60 years of age and in those with hypovolemia or pre-existing chronic kidney disease. Use of medications that may impair kidney perfusion or function may also be considered a risk factor for acute phosphate nephropathy [22]. These cases whereby high doses of oral sodium phosphate solution were ingested as a pre-operative or pre-procedure bowel cleansing preparation and caused harm in susceptible patients resulted in the abandonment of its availability in the U.S. As a result, smaller doses of oral phospho-soda solution were also not an available option during the national intravenous sodium phosphate shortage.

The greatest potential for an adverse effect of this therapy would be either diarrhea with intestinal discomfort or hypocalcemia. Phosphate therapy, in higher doses, is used for its laxative effect. The high osmolality with saline enema solution creates an osmotic gradient drawing water into the intestine, resulting in the development of a softer stool, which is easier for the patient to evacuate. However, we intentionally diluted the saline enema solution with water to reduce the potential for this therapeutic effect. Three patients, all from the lower dosage group, experienced diarrhea. Two of the patients received multiple doses of acetaminophen liquid, which is a known cause of diarrhea due to its high sorbitol content [23]. The third patient was receiving concurrent polyethylene glycol and senna for prophylaxis of opioid-induced constipation. Two patients among these patients were also receiving intravenous broad spectrum antibiotic therapy (cefepime) known to elicit diarrhea in 14% of patients. None of the patients experienced emesis and the highest gastric residual volume recorded was 180 mL. Unfortunately, it is difficult to ascertain whether phosphorus therapy caused diarrhea in these cases, as critically ill patients often require other medications known to induce diarrhea. Acute hypocalcemia and visceral calcification from substantial increases in the serum phosphorus concentration could occur if phosphorus therapy is given in excessive doses whereby a serum phosphorus-calcium product greater than 50 to 60 is exceeded [24]. Two patients had a significant fall in the serum iCa. However, hypocalcemia (serum iCa < 1.12 mmol/L) occurs in over 20% of critically ill trauma patients due to multiple metabolic factors [25] and it is unlikely that this enteral phosphorus therapy caused hypocalcemia, given the modest increases in the serum phosphorus concentration. Finally, each 34 mmol of phosphorus will provide 41 mEq (41 mmol) of sodium, which should be taken into consideration with the patients’ overall fluid and sodium intake.

It should be emphasized that this method for treating hypophosphatemia is not recommended for routine or long-term use as saline enema solution is not intended for enteral use. It was implemented as a temporary, short-term solution under the constraint of the lack of conventional enteral phosphorus preparations suitable for patients requiring high doses of phosphorus and who were receiving EN via an intragastric feeding tube. Further follow-up with additional phosphorus therapy was addressed via administration of intravenous potassium phosphate as the serum potassium concentrations declined and the primary trauma service was made aware of the preferred use of potassium phosphate in lieu of potassium chloride in the patient with concurrent hypokalemia and hypophosphatemia. Repeated phosphorus therapy for at least the first week [1,17,21] of nutrition therapy will be required for many critically ill patients, particularly in those experiencing refeeding syndrome [3]. These data could potentially be expanded to other patient populations; however, lower doses would probably be preferable as critically ill trauma and thermally injured patients have high requirements [1,17,21].

The strengths of this study are the homogeneity of the population and their presence in the TICU at a single Level I trauma center facilitating a similar level of patient care management among all the patients. Another strength is that the patients were studied near the initiation of EN at the height of their increased phosphorus requirements [3]. The limitations of this study include its retrospective study design, limited number of patients and inadequate power, and the fact that all the patients were from a single institution, which restricts its generalizability to other trauma centers.

## 5. Conclusions

Our data indicated that intragastric phosphate administration using a small volume of saline enema solution improved the serum phosphorus concentrations in most patients who required continuous EN. Those who received two 34 mmol doses of phosphate tended to respond better than those who received one dose. Minimal adverse effects were noted. This therapy is not recommended for routine clinical use, but it illustrates a temporary, short-term alternative solution to a national intravenous sodium phosphate shortage.

## Figures and Tables

**Table 1:** Patient characteristics.

Variable *	Lower Dose	Higher Dose	*p*
N	22	11	-
Age, y	49 [29, 64]	34 [27, 48]	0.331
Actual weight, kg	83 [68, 102]	82 [72, 90]	0.789
Ideal body weight, kg	65 (59, 75)	71 (66, 78)	0.292
Adjusted dosing weight, kg	76 [68, 85]	75 [72, 84]	0.619
Body mass index, kg/m^2^	26.3 [23.0, 33.1]	27.0 [24.5, 28.8]	0.717
Sex, male/female, n/n	14/8	9/2	0.430
Race			
Black, n	13	7	0.586
White, n	7	4	
Hispanic, n	2	0	
Admission diagnosis			
Motor vehicle collision, n	16	7	0.329
GSW/KSW, n	2	3	
Fall/assault, n	1	1	
Other, n	3	0	
Severe TBI with ICP monitoring, n (%)	7 (32%)	2 (18%)	0.681
Ventilator dependent, n	21 (95%)	11 (100%)	1.000
TICU length of stay, d	14 [9, 19]	14 [12, 18]	0.515
Hospital length of stay, d	28 [18, 45]	21 [19, 41]	0.390
Ventilator dependent, n (%)	21 (95%)	11 (100%)	1.000
Survived, n (%)	17 (77%)	10 (91%)	0.637
Serum potassium, mEq/L	4.2 [4.0, 4.3]	4.2 [4.0, 4.4]	0.546
mmol/L	4.2 [4.0, 4.3]	4.2 [4.0, 4.4]	
White blood cell count, cells/µm^3^	10.7 [9.3, 13.5]	10.5 [7.8. 14.3]	0.717
Serum magnesium, mg/dL	2.0 [2.0, 2.1]	1.8 [1.7, 2.1]	0.055
mmol/L	0.82 [0.82, 0.86]	0.74 [0.70, 0.86]	
C-reactive protein, mg/dL	25.0 [17.9, 34.3]	18.1 [10.9, 31.8]	0.172
mg/L	250 [179, 343]	181 [109, 318]	
Prealbumin, mg/dL	9.0 [4.5, 13.5]	10.0 [7.0, 14.3]	0.364
mg/L	90 [45, 135]	100 [70, 143]	
Serum creatinine, mg/dL	0.8 [0.6, 1.0]	0.8 [0.7, 0.9]	0.907
µmol/L	71 [53, 88]	77 [62, 80]	
Serum urea nitrogen, mg/dL	18 [13, 27]	18 [10, 27]	0.804
mmol/L	6.4 [4.6, 9.6]	6.4 [3.6, 9.6]	
Serum glucose, mg/dL	135 [113, 164]	113 [104, 122]	0.070
mmol/L	7.5 [6.3, 9.1]	6.3 [5.8, 6.8]	
Total fluid intake, L/d	2.9 [2.2, 3.5]	3.1 [1.8, 3.4]	0.954
Total fluid output, L/d	1.9 [1.4, 3.0]	2.2 [1.1, 3.7]	0.554
Received vasopressors during EN, n (%)	2 (9%)	1 (9%)	1.000

* d, day; GSW, gunshot wound; KSW, knife stab wound; ICP, intracranial pressure; TBI, traumatic brain injury; TICU, trauma intensive care unit.

**Table 2:** Response to enteral phosphorus therapy.

Variable *	Lower Dose	Higher Dose	*p*
Number of patients, n	22	11	-
Phosphorus dose, mmol	34	68	-
Phosphorus dose, mmol/kg dosing weight	0.45 [0.40, 0.50]	0.91 [0.81, 0.94]	0.001
EN phosphorus intake, mmol ¶	19.5 [8.8, 34]	24 [12, 33]	0.789
Day of EN, d ¶	2 [1, 7]	2 [1, 4]	0.556
TICU day, d ¶,	5 [3, 10]	4 [2, 6]	0.317
Caloric intake, kcals/d ¶	899 [423, 1405]	1035 [443, 1513]	0.717
Carbohydrate intake, g/d ¶	64 [39, 136]	107 [55, 161]	0.480
Insulin intake, units/d ¶	0 [0, 3]	0 [0, 0]	0.477
Arterial pH pre-dose	7.42 [7.36, 7.47]	7.42 [7.37, 7.45]	0.560
Arterial pH post-dose	7.45 [7.39, 7.49]	7.44 [7.29, 7.50]	0.830
Initial serum phosphorus, mg/dL	2.6 [2.4, 2.8]	2.5 [2.1, 2.8]	0.465
mmol/L	0.84 [0.77, 0.90]	0.81 [0.68, 0.90]	
Final serum phosphorus, mg/dL	2.8 [1.9, 3.3]	2.9 [2.2, 3.0]	0.878
mmol/L	0.90 [0.61, 1.07]	0.94 [0.71, 0.97]	
∆ in serum phosphorus, mg/dL	0.2 [−0.5, 0.7]	0.6 [−0.3, 0.8]	0.646
mmol/L	0.06 [−0.16, 0.23]	0.19 [−0.10, 0.26]	
Improvement in serum phosphorus, n (%)	12 (55%)	8 (73%)	0.436
Initial serum iCa, mmol/L	1.11 [1.07, 1.17]	1.14 [1.11, 1.15]	0.984
Final serum iCa, mmol/L	1.15 [1.09, 1.20]	1.12 [1.08, 1.16]	0.251
Diarrhea, n (%)	3 (14%)	0 (0%)	0.534

¶ on day of phosphorus therapy. * EN, enteral nutrition; iCa, ionized calcium concentration. TICU, trauma intensive care unit; ∆, change.

**Table 3:** Population characteristics of those who responded versus did not respond to oral phosphorus therapy.

Variable *	Responded	Not Responded	*p*
Number of patients, n	20	13	-
Initial serum phosphorus, mg/dL	2.6 [2.3, 2.8]	2.6 [2.3, 2.8]	0.811
mmol/L	0.84 [0.74, 0.90]	0.84 [0.74, 0.90]	
Final serum phosphorus, mg/dL	3.1 [2.9, 3.6]	1.9 [1.6, 2.3]	0.001
mmol/L	1.0 [0.94, 1.16]	0.61 [0.52, 0.74]	
Phosphorus dose, mmol	35 [35, 70]	35 [35, 53]	0.332
Phosphorus dose, mmol/kg dosing weight	0.53 [0.44, 0.85]	0.51 [0.42, 0.80]	0.495
EN phosphorus intake, mmol/d	16 [7, 31]	24 [16, 35]	0.172
Age, y	36 [26, 60]	48 [40, 66]	0.167
Body mass index (kg/m^2^)	27.5 [24.5, 31.5]	25.8 [22.6, 30.0]	0.585
Serum creatinine, mg/dL	0.8 [0.6, 0.9]	0.8 [0.8, 1.0]	0.202
µmol/L	71 [53, 80]	71 [71, 88]	
C-reactive protein, mg/dL ¶	25.0 [16.5, 34.3]	19.6 [10.0, 29.5]	0.144
mg/L ¶	250 [165, 343]	196 [100, 295]	
Prealbumin, mg/dL ¶	8.5 [5.8, 13.0]	10.0 [6.0, 16.0]	0.518
mg/L ¶	85 [58, 130]	100 [60, 160]	
WBC, cells/µm^3^	10.6 [9.0, 12.0]	11.9 [9.5, 15.8]	0.308
Arterial pH pre-dose	7.42 [7.36, 7.48]	7.40 [7.36, 7.44]	0.639
Arterial pH post-dose	7.44 [7.34, 7.47]	7.45 [7.41, 7.51]	0.208
Admit diagnosis of MVC, n (%)	15 (75%)	8 (62%)	0.745
Traumatic brain injury, n (%)	5 (25%)	4 (31%)	1.000
Survived, n (%)	16 (80%)	11 (85%)	1.000
Day of EN, d ¶	2 [1, 4]	2 [1, 8]	0.955
TICU day, d ¶,	4 [3, 6]	4 [2, 11]	0.741
Caloric intake, kcals/d ¶	703 [340, 1469]	985 [777, 1467]	0.261
Carbohydrate intake, g/d ¶	60 [28, 123]	108 [63, 162]	0.203
Serum glucose, mg/dL ¶	118 [109, 139]	136 [115, 161]	0.196

¶ on day of phosphorus therapy. * EN, enteral nutrition; MVC, motor vehicle collision; TICU, trauma intensive care unit; WBC, white blood cell count.

## Data Availability

The dataset used and analyzed in the current study is available from the corresponding author upon reasonable request.
